# Reconstruction of the Sternoclavicular Joint After Excessive Medial Clavicle Resection

**DOI:** 10.1016/j.eats.2021.08.020

**Published:** 2021-11-18

**Authors:** Marko Nabergoj, Alexandre Lädermann, Xueling Chong, Sidi Wang, Sean W.L. Ho

**Affiliations:** a Valdoltra Orthopaedic Hospital, Ankaran; b Faculty of Medicine, University of Ljubljana, Ljubljana, Slovenia; c Division of Orthopaedics and Trauma Surgery, La Tour Hospital, Meyrin; d Faculty of Medicine, University of Geneva, Geneva; e Division of Orthopaedics and Trauma Surgery, Department of Surgery, Geneva University Hospitals, Geneva, Switzerland; f Department of Orthopaedic Surgery, Tan Tock Seng Hospital, Singapore

## Abstract

Medial clavicle excision is a rarely indicated procedure and may be performed in different pathologies affecting the medial clavicle. Excessive medial clavicle resection with an injury to the costoclavicular ligament often leads to poor postoperative results. The exact surgical treatment used in this kind of pathology when conservative treatment is unsuccessful remains unclear. The aim of this Technical Note is to describe our preferred surgical technique to treat this condition.

Medial clavicle excision has been used as surgical treatment in cases of recurrent instability of the sternoclavicular joint (SCJ), posttraumatic arthrosis, osteomyelitis, tumor, as well as nonunion or irreparable comminution of the proximal part of the clavicle.^1^, ^2^, ^3^, ^4^ During resection of the medial clavicle, the key principle to obtaining a satisfactory postoperative clinical outcome is to avoid injury to the costoclavicular ligament (CCL). Damage to the CCL can result in SCJ instability and poor clinical results.^1^^,^^5^^,^^6^ The recommended extent of medial clavicular resection in the literature is variable, with one author proposing a resection of 4 cm.^1^^,^^6^, ^7^, ^8^, ^9^, ^10^, ^11^ However, cadaveric biomechanical studies have reported that the average distance from the inferior articular surface of the medial clavicle and the most medial clavicular insertion of the CCL is 1.1 cm to 1.26 cm.^12^^,^^13^ Additionally, a continuity between the capsule of the SCJ and the CCL, which has also been observed in a previous anatomical study by Cave,^14^ has been found in 3% to 12% of shoulders.^13^^,^^15^ Authors who reported the absence of free space between the CCL and the inferior part of the SCJ cautioned about the lack of a real safe zone for excision of the CCL and recommended identifying the CCL in all cases before resection was initiated.^12^^,^^15^

Excessive medial resection of the clavicle can result in a persistently unstable sternoclavicular joint. In such cases that have failed conservative treatment, surgical treatment is indicated. This Technical Note aims to describe our preferred surgical technique to treat this condition.

## Surgical Technique

### Preoperative Patient Positioning

Preoperative intravenous antibiotic is administered before the start of surgery. The surgical procedure is performed with the patient under general anesthesia in a semiseated position with a towel roll placed under the central spine so that the chest and SCJ protrude forward. The sites of iliac graft harvesting and the operative arm are prepped and draped so that the SCJ can be examined dynamically during the procedure.

### Iliac Bone Autograft Harvesting

**Fig 1:**
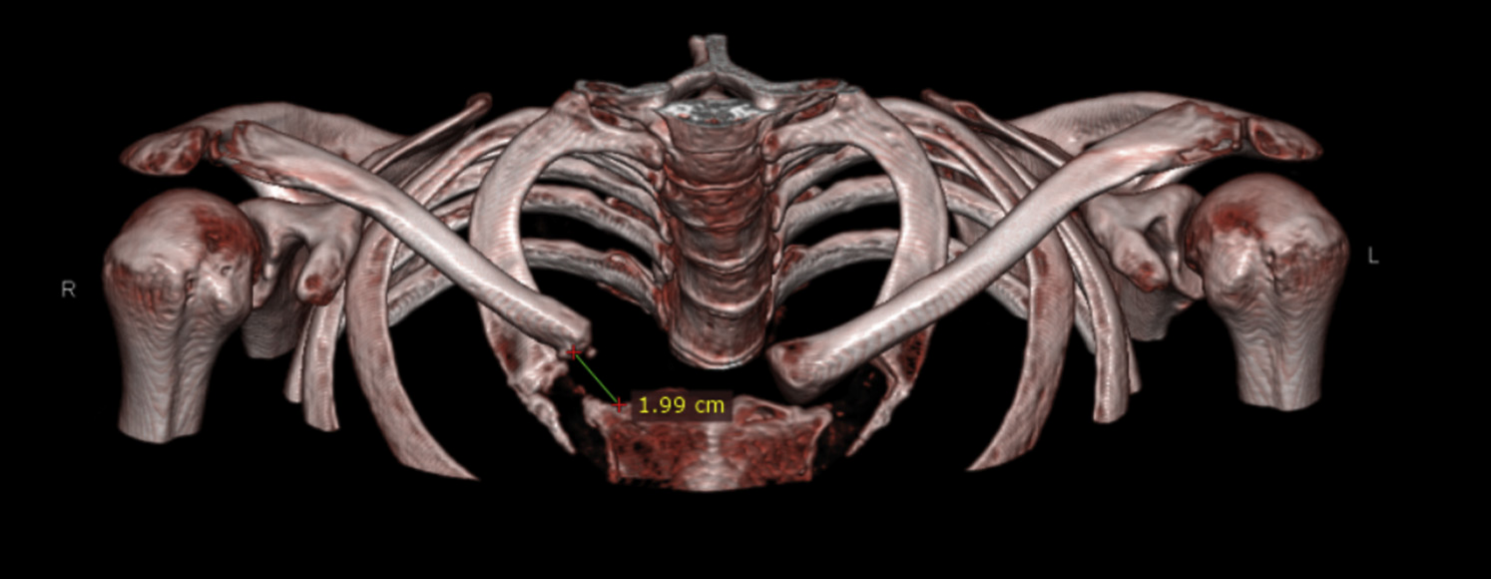
Three-dimensional reconstruction of the shoulder girdle from computed tomography. The defect after medial right clavicular resection is measured at 1.99 cm.

**Fig 2:**
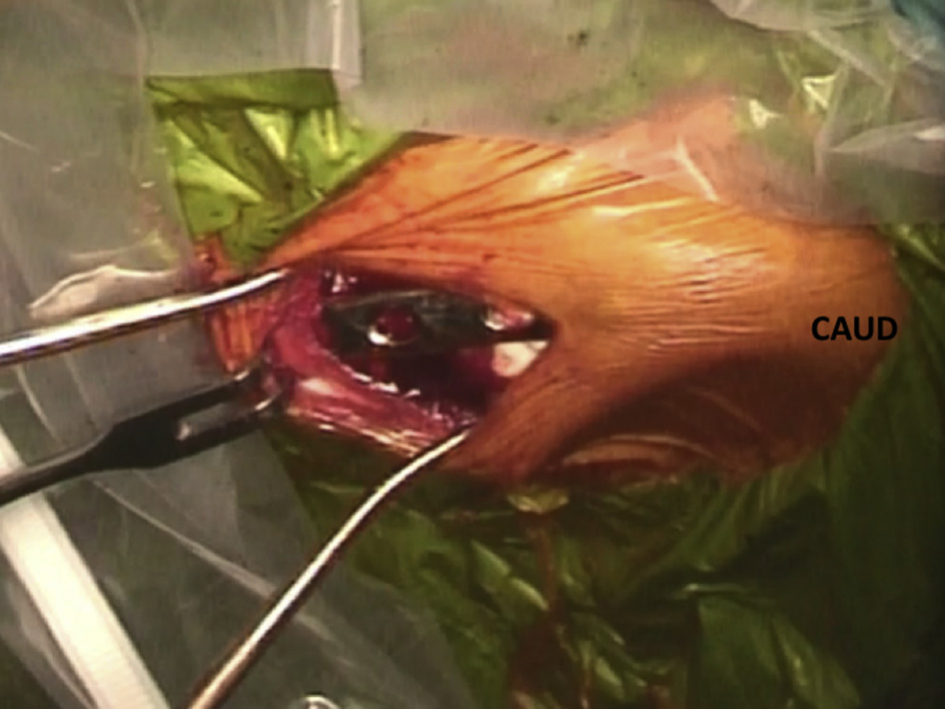
Patient is placed in a semiseated position. Iliac crest on the right side is seen. After iliac bone graft harvesting, a cosmetic deformity is a possibility. This is especially so in thin patients. To avoid it, we propose to fill the bone defect with an absorbable hemostatic gelatin sponge (Spongostan; Ferrosan, Søborg, Denmark) and additionally, to fix a 1/3 tubular plate on top of the iliac crest over the bone defect to allow the iliac crest to recover its shape. CAUD, caudal.

The bony landmarks of the anterior superior iliac spine (ASIS) and the iliac crest are palpated. A horizontal incision is performed from 3 cm posterior to the ASIS and extended posteriorly for 3 to 5 cm along the iliac crest. The incision length depends on the size of the autograft needed, based on the preoperative computed tomography (CT) measurements (Fig 1). The cortico-cancellous bone graft is harvested with the straight osteotome. First, 2 coronal cuts are made through the ilium. Thereafter, a horizontal cut is made, and the iliac crest bone autograft (ICBA) is obtained. The defect in the iliac crest is filled with an absorbable hemostatic gelatin sponge (Spongostan; Ferrosan, Søborg, Denmark) to decrease bone bleeding. In thin patients, a cosmetic deformity may be present after iliac bone harvesting. As such, we propose to secure a small plate on top of the iliac crest over the bone defect to prevent cosmetic deformity (Fig 2, Video 1).

### Surgical Approach Over the Clavicle and SCJ

**Fig 3:**
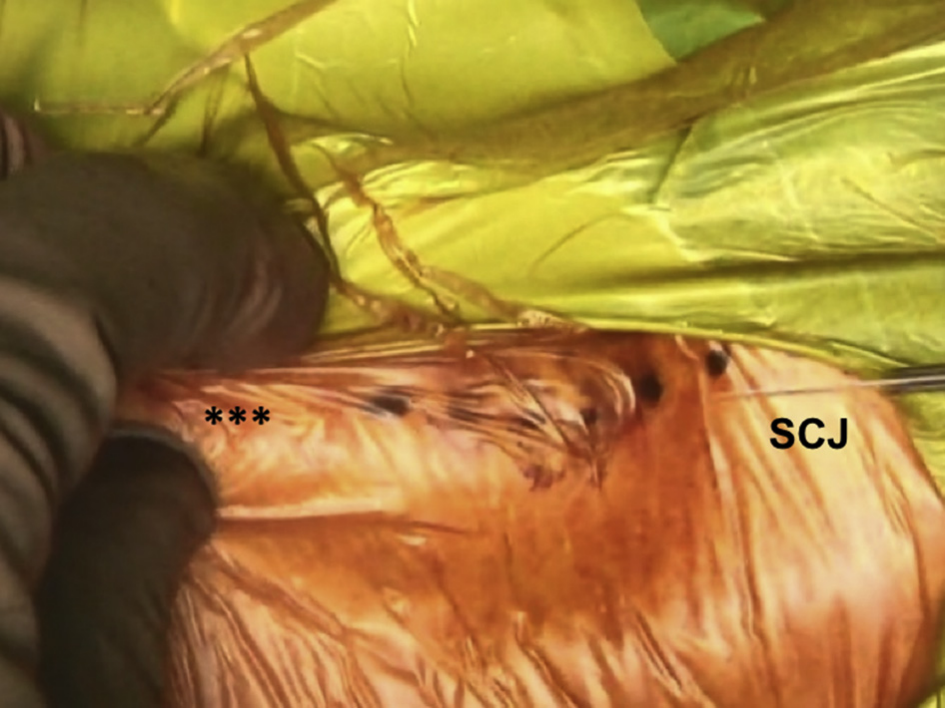
Patient is placed in a semi-seated position. The medial end of the right clavicula and the SCJ are palpated, and an incision starting at 3 cm laterally to the medial end of the clavicle and extended over the SCJ is marked with a surgical pen. ∗∗∗ - Clavicle shaft. SCJ, sternoclavicular Joint

**Fig 4:**
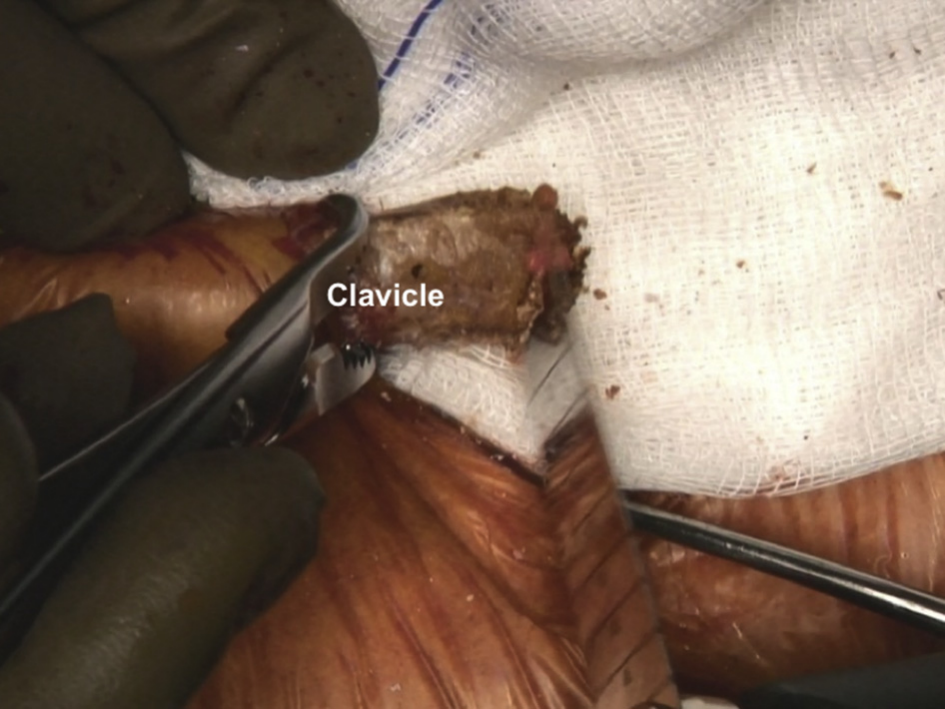
Patient is placed in a semi-seated position. The medial edge of the right clavicle is revitalized with a saw. A gauze is placed under the medial clavicle to prevent skin injury and bone debris from entering the surgical field.

**Fig 5:**
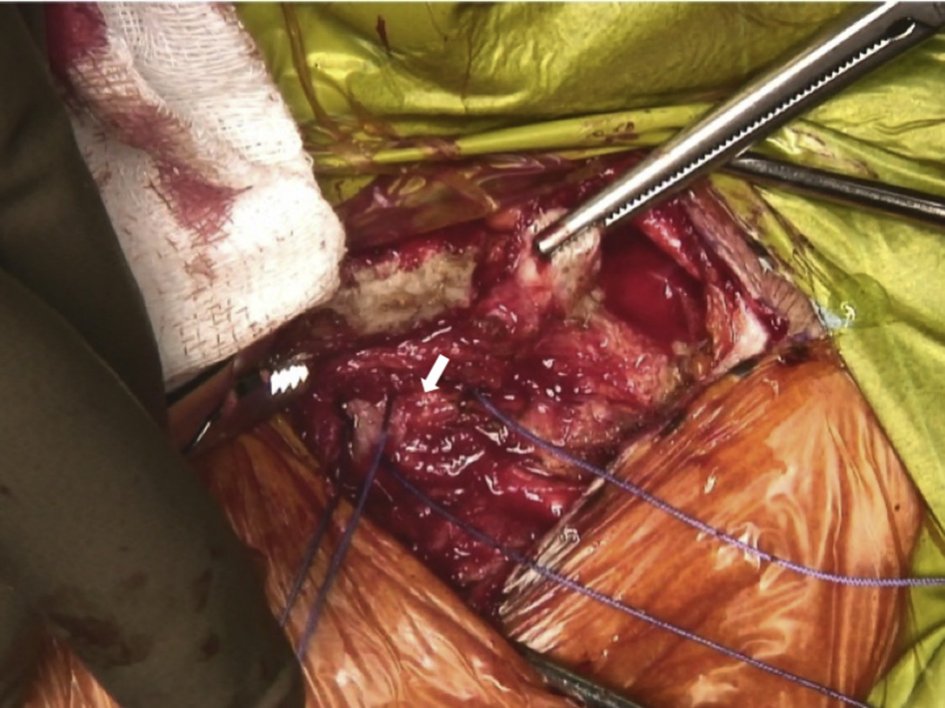
Patient is placed in a semi-seated position. Two strands of sutures (Ethibond 5; Ethicon, Somerville, NJ) are passed through the first rib (white arrow) under the medial part of the right clavicle for final costoclavicular stabilization.

**Fig 6:**
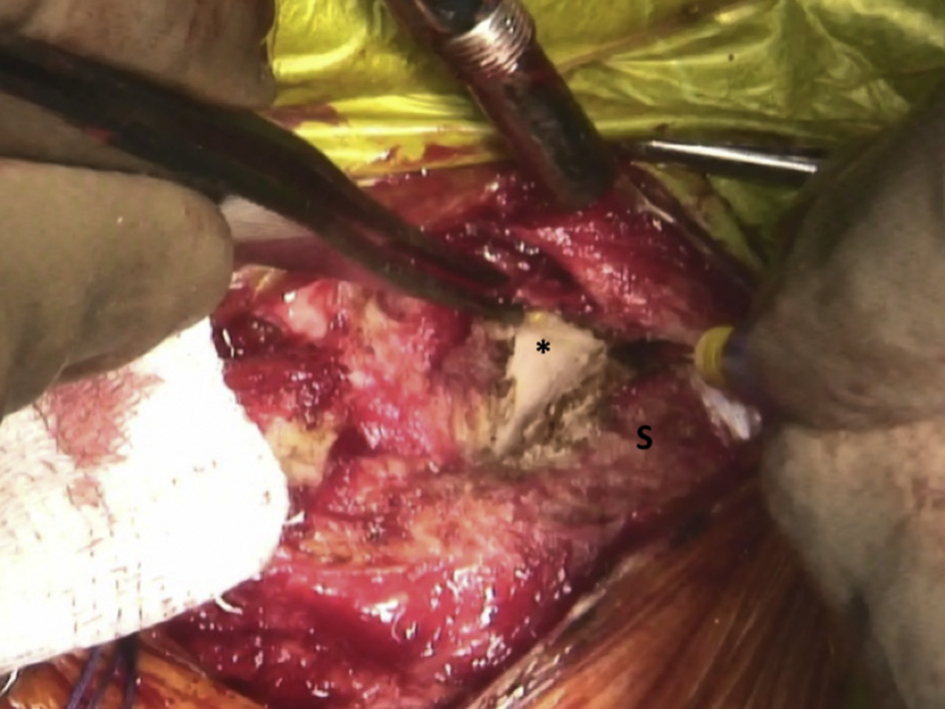
Patient is placed in a semi-seated position. The posterior capsule (∗) of the right SCJ is released from the sternum prior to drilling of the sternal tunnels. SCJ, sternoclavicular joint; S, sternum.

**Fig 7:**
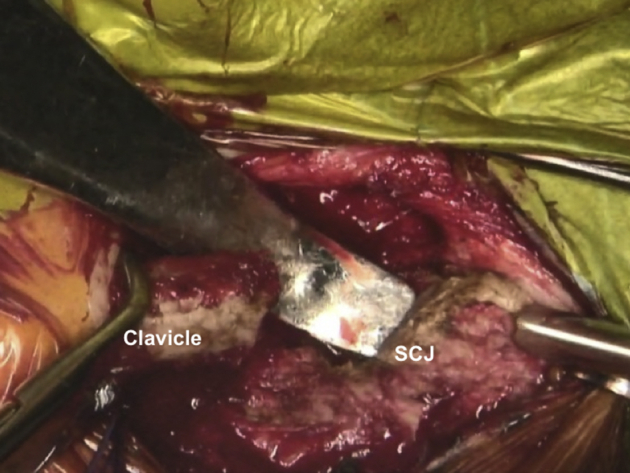
Patient is placed in a semi-seated position. Right SCJ is seen. Superior and inferior sternal tunnels are drilled in an oblique direction on the sternum from the anterior cortex towards its right articular surface in order to avoid an injury, whereas a malleable retractor is placed behind the sternum for additional protection. SCJ, sternoclavicular joint.

In view of the proximity of critical mediastinal and neurovascular structures, we recommend that a cardiothoracic surgeon is on standby during this relatively risky procedure. The medial end of the clavicle and the SCJ is palpated and an incision line is drawn starting 3 cm lateral to the medial clavicular end and extended over the SCJ (Fig 3, Video 1). After the initial incision, the platysma is identified and preserved for later repair. A careful dissection of the proximal clavicular remnant, the upper part of the first rib and a thorough release of the anterior capsule of the SCJ are performed so that they are easily accessible for later reconstruction. The medial clavicular edge is revitalized with a saw (Fig 4, Video 1). Two high-strength sutures (Ethibond 5; Ethicon, Somerville, NJ) are passed through the first rib under the medial clavicle for final costoclavicular stabilization (Fig 5, Video 1). The posterior SCJ capsule is then slightly released (Fig 6, Video 1). A superior and inferior sternal tunnel of 4 mm diameter are drilled in an oblique direction on the sternum from the anterior cortex towards its right articular surface, while a malleable retractor is placed behind the sternum to prevent iatrogenic injury (Fig 7, Video 1).^16^

### Preparation of the Gracilis Allograft Tendon (GAT) and ICBA

**Fig 8:**
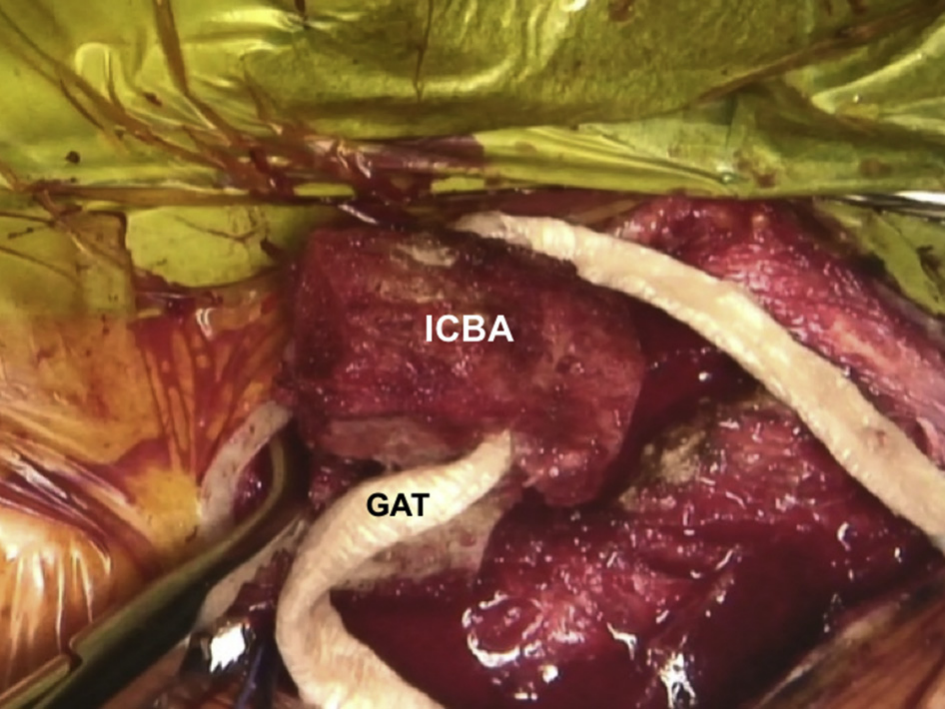
Patient is placed in a semi-seated position. Right SCJ is seen. Prepared GAT is passed through a hole drilled horizontally through the ICBA. SCJ, sternoclavicular joint; GAT, gracilis allograft tendon; ICBA, iliac crest bone autograft.

The 4.0 mm diameter GAT is tagged with a stitch of approximately 2 to 3 cm in length on the free ends with a nonabsorbable suture material (Ethibond 6; Ethicon). The ICBA is shaped with a rongeur such that it nicely fits between the medial end of the clavicle and the sternum, while avoiding overstuffing. A tunnel is drilled horizontally through the ICBA, and the prepared GAT is passed through it (Fig 8, Video 1).

### Reconstruction of the Medial Clavicle With Iliac Crest Bone Autograft

**Fig 9:**
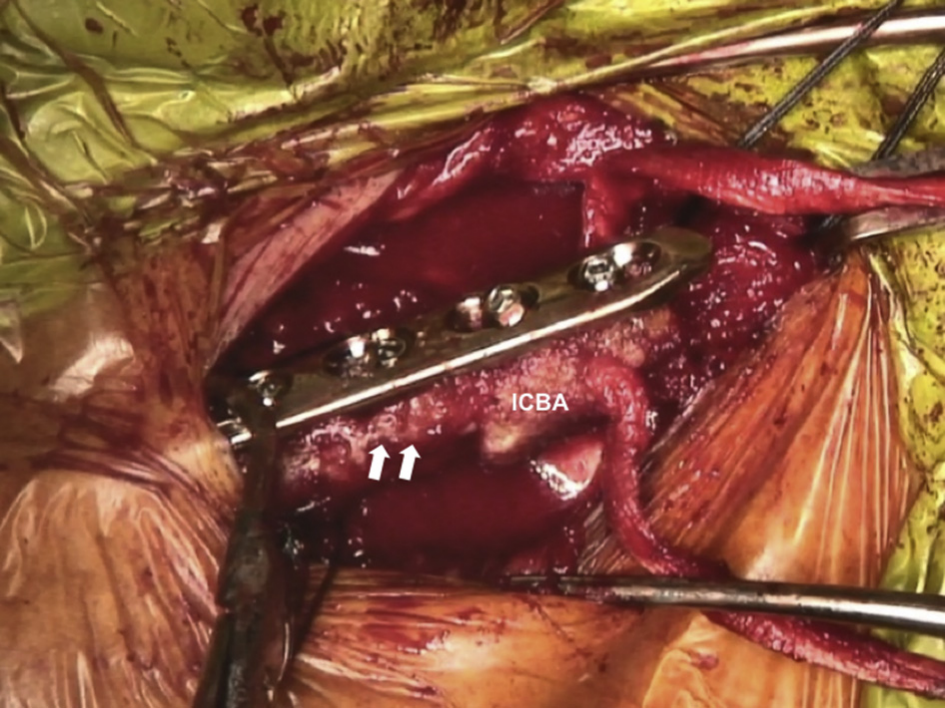
Patient is placed in a semi-seated position. The ICBA is fixed on the medial end of the right native clavicle (white arrows) with a 3.5mm LC-DCP. ICBA, iliac crest bone autograft; LC-DCP, low contact dynamic compression plate.

**Fig 10:**
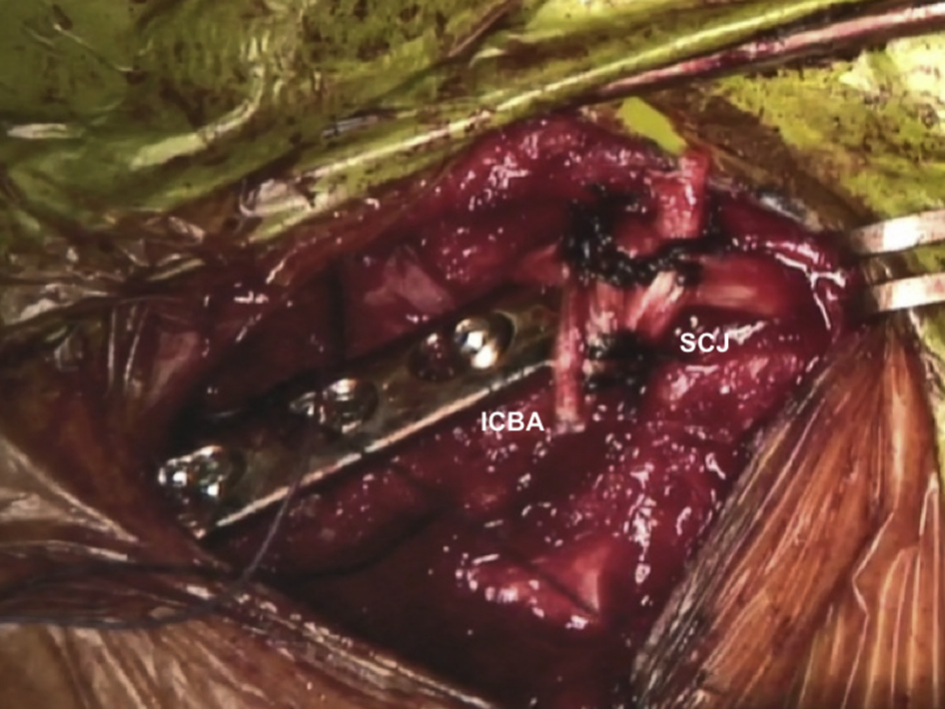
Patient is placed in a semi-seated position. Stabilization of the right SCJ with a GAT, that is passed through the ICBA and sternal tunnels and secured with a high-strength suture (Ethibond 6; Ethicon, Somerville, NJ), using the figure-of-eight technique. SCJ, sternoclavicular joint; GAT, gracilis allograft tendon; ICBA, iliac crest bone autograft.

**Fig 11:**
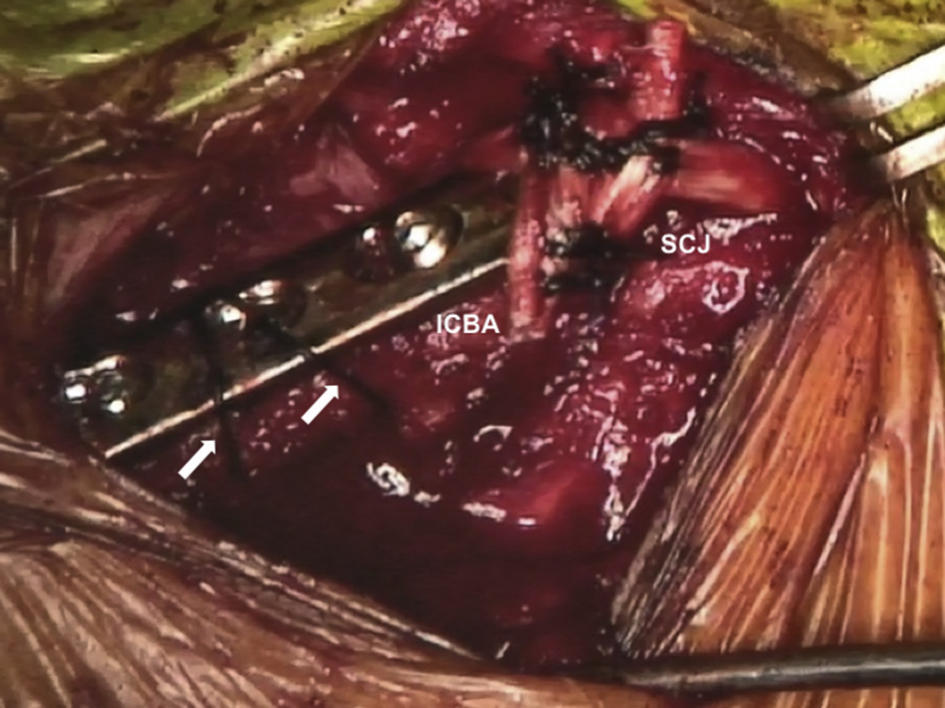
Patient is placed in a semi-seated position. Right reconstructed SCJ is seen. The final costoclavicular stabilization reconstructing the CCL is performed with the high-strength sutures (Ethibond 5; Ethicon, Somerville, NJ) passing through the first rib and around the medial native clavicle with a fixed plate (white arrows). SCJ, sternoclavicular joint; CCL, costoclavicular ligament.

The ICBA is fixed onto the medial end of the clavicle with a plate (Fig 9, Video 1). Afterward, the GAT, which is passed through the ICBA, is pulled by shuttle sutures through the bone tunnel in the sternal part of the SCJ so that it forms a figure-of-eight configuration. While the joint is reduced, stabilization of the SCJ is performed by suturing both free ends of the GAT to one another using the high-strength suture (Ethibond 5) (Fig 10).^17^ The figure-of-eight reconstruction is chosen because it is the stiffest construct for SCJ instability repair.^18^ The final costoclavicular stabilization reconstructing the CCL is performed with the high-strength sutures passing through the first rib. Each strand of the sutures is brought around the opposite sides of the plate, and multiple knots are performed on the superior surface (Fig 11, Video 1). The SCJ can be dynamically tested for stability. During the procedure, the posterior structures should be protected with a Hohmann retractor.

### Closure

**Fig 12:**
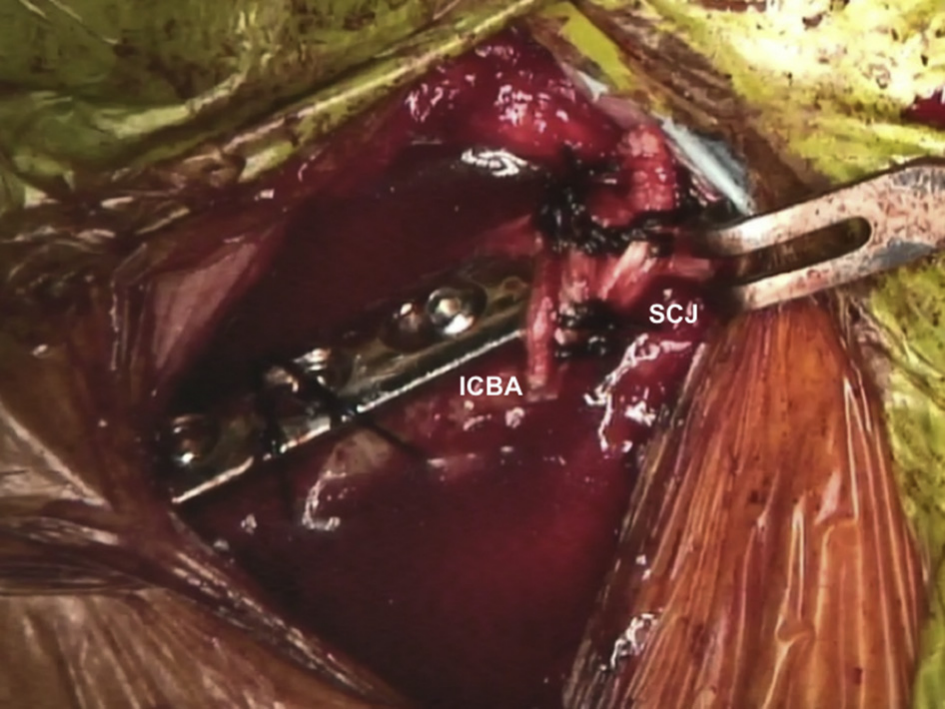
Patient is placed in a semi-seated position. Reconstructed right SCJ is seen. At the end of the surgery, pooled saline is left inside the surgical field and observed for 10 to 20 seconds. If air bubbles are seen coming up from underneath the clavicle, there has been a perforation of the pleura or lung. ICBA, iliac crest bone autograft; SCJ, sternoclavicular joint.

The surgical field is irrigated with saline solution. Saline solution is allowed to pool inside the surgical field, and the anesthetist performs a Valsalva maneuver for the patient (Fig 12, Video 1). If air bubbles are seen coming up from underneath the clavicle, there has been a perforation of the pleura or lung. Next, the joint capsule and the platysma are reconstructed with Vicryl 2 sutures. Last, the subcutaneous layer and skin are closed in a standard fashion.

Table 1 explains pearls and pitfalls of this surgical technique. Table 2 explains advantages and disadvantages of this surgical technique.

**Table 1:** Pearls and Pitfalls

Pearls
The cosmetic defect after autograft harvesting from the iliac crest can be avoided by filling the defect with an absorbable hemostatic gelatin sponge (Spongostan; Ferrosan, Søborg, Denmark) and securing a 1/3 tubular plate over the iliac crest.
Injury of the retro-sternoclavicular joint structures is minimized during drilling of the sternal tunnels by drilling in an oblique direction on the sternum from the anterior cortex toward its articular surface and by placement of a malleable retractor behind the sternum.
The autograft is shaped to fit between the medial clavicle edge and the sternum. This should be done appropriately to avoid overstuffing.
While the joint is reduced, the stabilization of the sternoclavicular joint is performed by suturing the free ends of the gracilis allograft tendon to one another using a high-strength suture.
After the reconstruction is finished, the saline is left inside the surgical field and observed for 10 to 20 seconds. Pneumothorax is excluded by absence of rising air bubbles.
Pitfalls
Postoperative nonunion or resorption of bone graft can occur that may require revision surgery.

**Table 2:** Advantages and Disadvantages of Our Technique

Advantages
This anatomic sternoclavicular joint reconstruction provides better restoration of shoulder mechanics and function, compared to nonanatomic soft tissue only reconstruction techniques.
Bone allograft is not used, which lowers the expenses and increases the potential for graft integration with the native clavicle.
Disadvantages
Demanding surgical technique, which results in longer operating time compared to soft tissue–only reconstruction techniques.
Surgical risks are present because of the proximity of important mediastinal and neurovascular structures.
Gracilis tendon allograft is used, which increases the expenses and decreases the potential for graft healing.
This procedure should only be used in patients with a low risk of osteosynthesis failure, including patients who are compliant to immobilization treatment, have an intact first rib, and do not smoke.

### Postoperative Protocol

The patient was admitted to the hospital for overnight observance. The operated upper extremity should be in a sling for 6 weeks after operation. Supervised passive range of motion in the scapular plane is permitted. After 6 weeks, the patient begins with a progressive rehabilitation program.

## Discussion

Resection of the medial clavicle is an infrequent surgical procedure and, if inadequately performed, results in poor clinical outcomes mandating further management.^1^^,^^5^^,^^6^ If conservative treatment fails, surgical treatment is proposed. However, due to the rarity of this condition, controversy remains regarding the ideal surgical technique that should be used in symptomatic SCJ instability after excessive medical clavicle resection.

**Fig 13:**
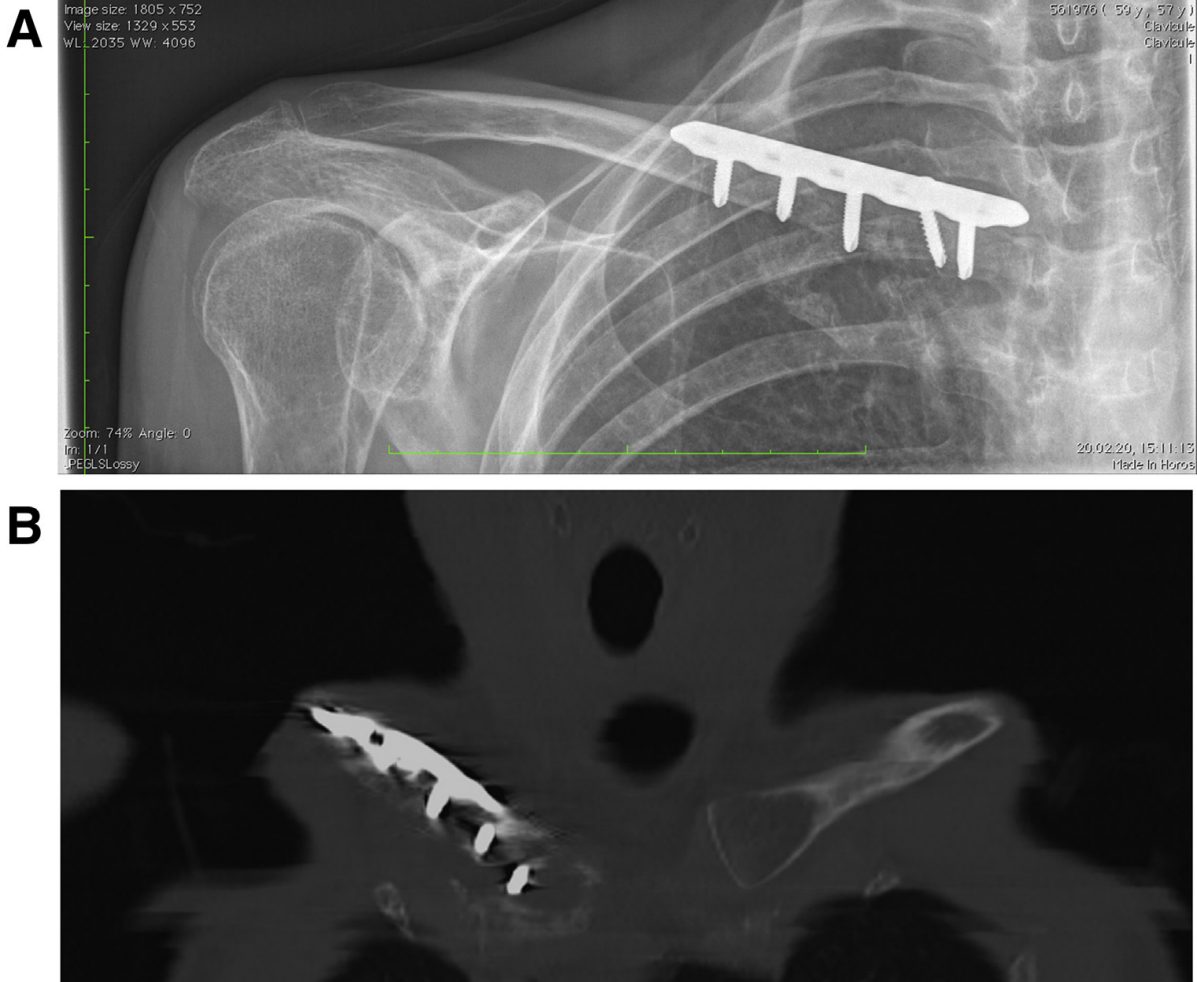
Repeat AP radiograph of the right clavicula (A) at eight weeks and CT (coronal view) of the right clavicula (B) at 1 year after operation show bony consolidation of the bone graft with restoration of the clavicle length. AP, anteroposterior; CT, computed tomography.

Some authors have previously described surgical options in cases of nonunion or comminuted fractures of the medial proximal clavicle. Dion et al.^2^ proposed to treat a medial clavicular fracture nonunion with medial clavicle excision combined with stabilization of the medial clavicle to the sternum with a figure-of-eight stabilization construct of palmaris longus graft. A similar solution was proposed by Sanchez et al.^19^ who described a stabilization of SCJ with a tibial anterior allograft after medial clavicle excision of the medial comminuted clavicle fracture. Although these soft-tissue reconstructions could be an option in previously described conditions with a high-risk of osteosynthesis failure, we believe that the procedure we proposed is more beneficial because it includes bony reconstruction in addition to soft tissue reconstruction. By reconstructing the bone, there is better anatomic reconstruction with improved shoulder mechanics and function restoration. In addition, with bony consolidation of the graft to the native clavicle (Fig 13), there is a lower risk of failure of the soft tissue reconstruction. Thus this technique should be used as the first option in the surgical treatment of a shortened clavicle because of excessive medial clavicle resection.

In cases where reconstruction of the normal clavicular osseous anatomy is unattainable, a salvage procedure such as a complete claviculectomy may be performed to try to ease symptoms.^20^ However, this should be achieved as a last possible surgical option, only when all other options are exhausted.

The type of surgical technique we propose in this article should only be used in patients with a low risk of osteosynthesis failure. This includes patients who are compliant to immobilization treatment, have an intact first rib, which is needed to recreate the CCL effect, and do not smoke. Not following these recommendations could lead to failure.^21^ To our knowledge, this is the first description in the literature reporting an anatomic reconstruction involving bone and ligamentous restoration of the medial clavicle and SCJ in case of excessive medial clavicle resection. Further long-term studies can be performed to assess whether this surgical reconstruction and stabilization technique adequately restores normal shoulder mechanics and function.

## Conclusion

In the setting of persistent sternoclavicular instability after excessive medial clavicle resection, a combination of osseous and soft tissue reconstruction can adequately restore sternoclavicular stability.

## Supplementary Data

ICMJE author disclosure forms

Video 1 Surgical technique demonstration of reconstruction of the SCJ after excessive medial clavicle resection. An iliac crest autograft is first harvested. The size of the graft is determined by the size of the clavicle defect. A 1/3 tubular plate can be applied after graft harvest onto the iliac crest to prevent cosmetic deformity. An incision is made over the medial clavicle and sternum, and the medial clavicle is exposed. It is revitalized with an oscillating saw and rongeur. The sternal portion of the SCJ is exposed, preserving the capsule. The first rib is exposed and two strands of Ethibond 5 are passed through it. Two tunnels are drilled from the anterior sternal edge to the sternal articular surface. The iliac crest bone graft is shaped to the defect size, with a horizontal tunnel drilled through it to pass the gracilis allograft. The iliac crest bone graft is fixed to the native clavicle with a 3.5mm limited-contact dynamic compression plate. The sutures from the first rib are passed around the clavicle. The gracilis allograft ends are introduced into the sternal tunnel in a figure-of-eight configuration. The joint is reduced, and the graft is sutured onto itself. The sutures from the first rib are tied around the clavicle for the final costoclavicular stabilization. Pooled saline solution is used to check for pneumothorax. The capsule and platysma are approximated and closed.
